# Role of radiofrequency ablation and cement injection for pain control in patients with spinal metastasis

**DOI:** 10.1186/s12891-021-04799-0

**Published:** 2021-10-29

**Authors:** Serhat Yildizhan, Mehmet Gazi Boyaci, Usame Rakip, Adem Aslan, Ihsan Canbek

**Affiliations:** Department of Neurosurgery, Afyonkarahisar Health Sciences University Faculty of Medicine, Dörtyol Neighb, 2078 st. No. 3/4, Afyonkarahisar, Turkey

**Keywords:** Vertebral metastasis, Radiofrequency ablation, Vertebroplasty, Palliative treatment

## Abstract

**Background:**

The study aimed to investigate the effects and reliability of simultaneous vertebroplasty and radiofrequency ablation or radiofrequency ablation applied alone for pain control in patients with painful spine metastasis, and to investigate the effect of preventing tumor spread in long-term follow-up.

**Methods:**

Patients with painful vertebrae metastasis in the Afyonkarahisar Health Sciences University, Medical Faculty, Hospital Neurosurgery Clinic between 01.01.2015 and 01.06.2020 were recruited. They were divided into groups according to the surgical procedures applied. Group 1 included 26 patients who underwent radiofrequency ablation only, and group 2 included 40 patients who underwent vertebroplasty with radiofrequency ablation. Computed tomography and magnetic resonance imaging were performed in all patients pre-operation. The patients were followed for at least 6 months. Magnetic resonance imaging was performed at the end of the 6th month in neurologically stable patients. The metastatic lesion, pain, and quality of life were evaluated with Visual Analog Scale and Oswestry Disability Survey before and after the procedure.

**Results:**

The mean VAS score before the procedure was 8.3 ± 1.07 in the RFA group, and a statistically significant difference was observed in VAS scores at all post-procedural measurement time-points (*p* < 0.001). The pain scores decreased at a rate of 58.8 and 69.6% of patients showed significant improvements in the QoL in the RFA-only group. The mean VAS score was 7.44 ± 1.06 in group RFA + VP before the procedure; the difference in the mean VAS scores was statistically significant at all measurement time-points after the procedure (*p* < 0.001). The mean pre-treatment Oswestry Index (to assess the QoL) was 78.50% in the RFA + VP group, which improved to 14.2% after treatment.

**Conclusion:**

Ablation + vertebroplasty performed to control palliative pain and prevent tumor spread in patients with painful vertebral metastasis is more successful than vertebroplasty performed alone.

## Introduction

Spinal metastases are common in patients with advanced malignancies, with a reported prevalence of 30% [1]. An autopsy series conducted by Scutellari et al. reported a prevalence of up to 70% [2]. Most metastases are detected in the thoracic spine (70%), followed by the lumbar spine (20%), and cervical and sacral spine (10%) [3]. The incidence of spinal metastasis has increased due to the aging population, increased life expectancy, and advancements in medical technology. The treatment strategy for patients with advanced-stage cancer is based on their health status or physical performance. The management of patients with expected survival periods < 3 months mainly comprises conservative or supportive care [4]. Surgical options are considered in cases where the survival expectation is longer. The most common tumours that metastasize to the spine include lung, breast, kidney, prostate, thyroid, and colorectal cancers and melanomas, myelomas, and lymphomas [5, 6]. The metastatic spread of tumours to the spine causes serious neurological problems arising from severe pain, spinal fractures, and compression of the nerve roots and spine by the lesion mass [7]. Radiotherapy, chemotherapy, isotopic therapy, bisphosphonate therapy, pharmacotherapy, radiofrequency ablation (RFA), and palliative surgery may be used for the treatment of spinal metastases [8]. RFA entails the use of a high-frequency alternating current that passes from the electrode needle to adjacent tissues, causing friction heating and tissue necrosis, including necrosis of metastatic tumour cells that produce nerve-stimulating cytokines and adjacent sensory nerve fibres (including those involved in sensory and pain conduction); additionally RFA arrests bone damage, inhibit pain-inducing osteoclastic activity, and also promotes the release of different cytokines and biochemical factors [9]. Ablation provides curative treatment for benign and malignant lesions measuring up to 3 cm. It reduces the tumour burden and mass effect on adjacent organs by reducing pain and effecting local disease control. Therefore, vertebroplasty (VP) should be performed after RFA, depending on lesion size and location, since the stability of the vertebral column is compromised [10]. This study evaluated the efficacy and reliability of simultaneous VP and RFA for the palliative management of patients with metastatic vertebral metastases with consideration of the current knowledge on this subject.

## Methods

The study was approved by the Afyonkarahisar Health Science University of Clinical Research Ethics Committee (dated 04.10.2019, approval number: 2019/310). Informed consent was obtained from all participants participating in the study and these consents were presented to the ethics committee. Eighty-eight patients with spinal metastasis were examined at the neurosurgery clinic of Afyonkarahisar Health Sciences University Hospital between 1 January, 2015 and 1 June, 2020. Among these patients, 66 patients who met the criteria and had a life expectancy of 3 months were included in the study. Patients with osteolytic vertebral metastasis, analgesic-resistant pain, an age of > 18 years, and with ≥3 months of life expectancy were included in the study. These patients had spinal involvements and were neurologically stable. The primary tumour was under control and their only complaint was pain. Patients with active primary tumors, neurologically unstable patients who had previously received radiotherapy for the spine, and patients in need of surgical decompression and instrumentation were excluded from the study.

**Fig. 1:**
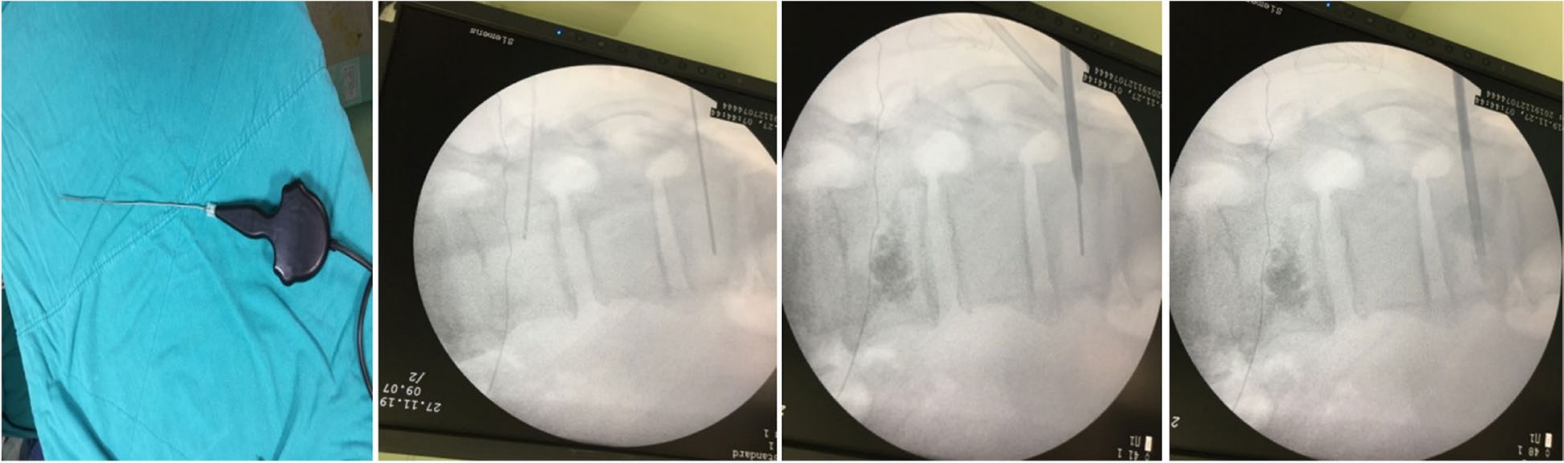
RF and cement application in metastatic involvement

**Fig. 2:**
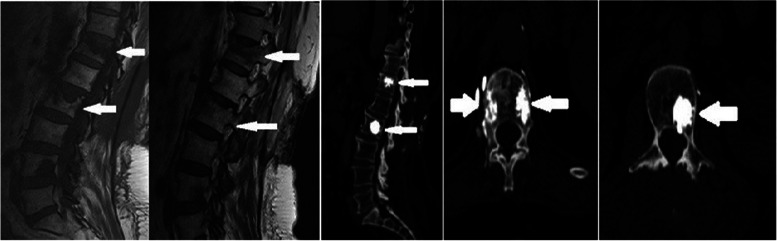
Sixty-four years old female patient. T12 and L2 vertebra metastatic involvement MRG images (preoperatively) and CT images after RFA and vertebroplasty

Sixty six of the eighty-eight patients met our eligibility criteria. Of these, 26 patients with middle and posterior element fracture out of the corpus underwent only RFA (group 1) and the remaining 40 patients underwent simultaneous treatment with VP and RFA (group 2). All patients underwent computed tomography (CT) and magnetic resonance (MR) imaging before the respective procedures. The Visual Analogue Scale (VAS; 0 = no pain, 10 = worst pain imaginable) was used for pain assessment. The Oswestry Disability Index was used to assess the quality of life (QoL). Participants were followed-up for up to 6 months. Patients were checked at 1, 3 and 6 months. Spinal CT was performed in patients with pain complaints during the control. Decompression surgery was performed in patients with 50% collapse of the vertebral corpus or fragments in the spinal canal. MR imaging was performed at the end of 6 months. RFA was administered to the vertebrae infiltrated by the neoplastic lesions, under local anaesthesia and conscious sedation at an average temperature of 90 °C for 2–4 min. Twenty six patients with vertebral metastases underwent VP with polymethylmethacrylate (PMM) radio-opaque bone cement injection after RFA (Figs. 1 and 2). Cardiovascular and respiratory parameters were monitored throughout the procedures, and preoperative and postoperative CT scans were compared.

### RFA and VP technique

The patients were placed in the pure prone position. Intravenous cephazolin was administered as a prophylactic antibiotic. After local anesthesia, a 10–15 G VP cannula was placed under the guidance of a Siemens brand c-arm fluoroscopy (1% lidocaine). The VP cannula was sent with the guide from the vertebral pedicle to the corpus border with the help of fluoroscopy. The guideline was removed at the beginning of the vertebral corpus. The VP cannula, which was hollow up to the middle of the vertebral corpus, was advanced with the help of a fluoroscopy. Then, the VP cannula was removed and the bone fragments accumulated in it were removed and sent to pathology. Using the same entrance localization, the VP cannula was advanced to the middle of the vertebral corpus with the help of fluoroscopy. RFA catheter was sent through the cannula.

RFA was conducted for an average duration of 3 min (2–4 min) at a temperature ranging from 90 °C with the help of the needle. The RFA catheter was removed and the prepared cement was injected into the cannula. Intermittent fluoroscopic examinations were conducted during cement injection to detect epidural leakage, eliminate lateral leakage, and evaluate cement distribution.

### Statistical analysis

The general linear model was used for statistical analysis of postoperative outcomes and 6-month follow-up outcomes for each group. The Mann-Whitney U test was used for comparisons between the two groups. Differences were considered significant if *p* < 0.05.

## Results

Group 1 consisted of 26 patients who underwent RFA only and group 2 of 40 patients who underwent VP with RFA. In group 1, decompression surgery was performed when vertebra collapsed at the end of the 1st month in 3 patients, at the end of the 3rd month in 4 patients and at the end of the 6th month in 3 patient. In group 2, none of the vertebrae treated during the 6-month follow-up period revealed tumour metastasis that could lead to advanced collapse and compression of the spinal cord.

Biopsy was negative in 6 patients in group 1 and 8 patients in group 2.

PMM leakage was observed from the posterior elements of the corpus vertebrae of the spinal canal in 4 patients; however, no interventions were required because the patients were asymptomatic.

Multiple myeloma was the most common primary malignancy and was observed in 22 patients (8 patients were group 1, 14 patients were group 2). Twenty eight patients had thoracic metastases, and eighteen with lumbar metastases. Multiple metastases were observed in 20 patients.

**Fig. 3:**
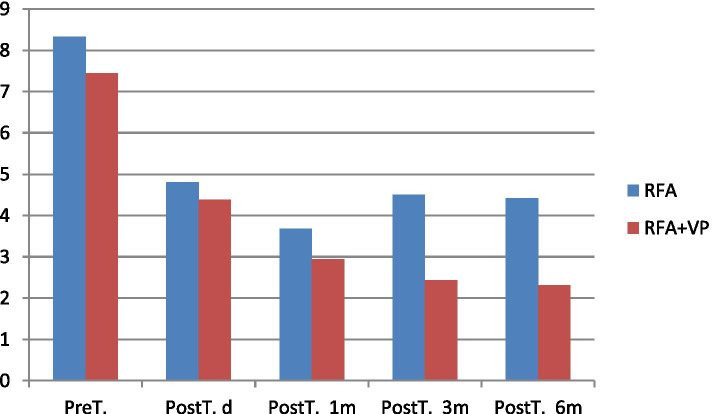
VAS score comparison between groups (VAS: Visual Analog Scala, RFA: Radiofrequency ablation, RFA + VP:Radiofrequency ablation+Vertebroplasty, Pre T.:Pre-treatment, PostT. d: Post-treatment one day, PostT 1 m, 3 m, 6 m: Post-treatment one month, three month, six month)

**Fig. 4:**
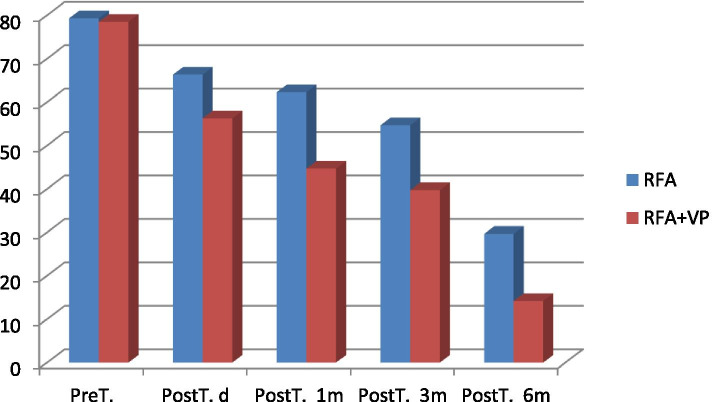
Oswestry score comparison between groups (RFA: Radiofrequency ablation, RFA + VP: Radiofrequency ablation+Vertebroplasty, Pre T.:Pre-treatment, PostT. d: Post-treatment one day, PostT 1 m, 3 m, 6 m: Post-treatment one month, three month, six month)

The mean VAS score before the procedure was 8.3 ± 1.07 in the RFA group (Table 1, Fig. 3), and a statistically significant difference was observed in VAS scores at all post-procedural measurement time-points (*p* < 0.001). The VAS score decreased significantly in the first 24 h after treatment and was determined to be 4.8 ± 1.03 in patients who underwent only RFA. The mean VAS scores obtained 1 and 6 months after the procedure exhibited statistically significant differences (*p* < 0.001). The VAS score, which started to increase in month 3 of treatment (4.50 ± 1.57), was 4.42 ± 1.08 points at the end of month 6. The mean pre-treatment Oswestry Disability Index (used to assess the QoL) was 79.33%, and significantly improved to 29.67% after treatment; the differences in the Oswestry Disability Index were statistically significant at all the measurement time-points after the procedure(*p* < 0.001) (Table 2, Fig. 4). The pain scores decreased at a rate of 58.8, and 69.6% of patients showed significant improvements in the QoL in the RFA-only group. The severity of pain increased and the vertebral corpus height decreased in 5 patients in this group during the 3-month follow-up; subsequently these patients underwent instrumentation surgery.

**Table 1:** VAS score comparison between groups

Group	PreT.	PostT. d	PostT. 1 m	PostT. 3 m	PostT. 6 m	***P*** value
**RFA**	8,33 ± 1,07^a^	4,80 ± 1,03^a,b,c^	3,67 ± 1,07^a,b^	4,50 ± 1,57^a^	4,42 ± 1,08^a,c^	a = *p* < 0.001 b = *p* < 0.001 c = *p* < 0.05
**RFA + VP**	7,44 ± 1,06^a^	4,38 ± 1,00^a,b^	2,94 ± 1,04^a,b,c^	2,44 ± 1,61^a,b,c^	2,31 ± 1,42^a,b,c^	a = *p* < 0.001 b = *p* < 0.001 c = *p* < 0,05

*VAS* Visual Analog Scala, *RFA* Radiofrequency ablation, *RFA + VP* Radiofrequency ablation+Vertebroplasty, *Pre T* Pre-treatment, *PostT. d* Post-treatment one day, *PostT 1 m, 3 m, 6 m* Post-treatment one month, three month, six month

^a^According to Pre-T among others

^b^According to PostT.d among PostT. 1m,PostT. 3m,PostT. 6m

^c^According to PostT.1m among PostT. 3m,PostT. 6m

**Table 2:** Oswestry score comparison between groups

Group	PreT.	PostT. d	PostT. 1 m	PostT. 3 m	PostT. 6 m	***P*** value
**RFA**	79,33 ± 3,75^a^	66,33 ± 6,26^a,b,c^	62,28 ± 6,32^a,b^	54,67 ± 11,86^a,b,d^	29,67 ± 8,77^a,c,d^	a = *p* < 0.001 b = *p* < 0.005 c = *p* < 0.001
**RFA + VP**	78,50 ± 5,20^a^	56,25 ± 9,66^a,b,c^	44,68 ± 10,34^a,b^	39,75 ± 16,09^a,b,d^	14,20 ± 12,32^a,c,d^	a = *p* < 0.001 b = *p* < 0.05 c = *p* < 0,001 d = *p* < 0.001

*RFA* Radiofrequency ablation, *RFA + VP* Radiofrequency ablation+Vertebroplasty, *Pre T*. Pre-treatment, *PostT. d* Post-treatment one day, *PostT 1 m, 3 m, 6 m* Post-treatment one month, three month, six month

^a^According to Pre-T among others

^b^According to PostT.d among PostT. 1m,PostT. 3m,

^c^According to PostT.d among PostT. 6m

^d^According to PostT. 3m among PostT 6m

The mean VAS score was 7.44 ± 1.06 in group RFA + VP (Table 1, Fig. 3) before the procedure; the difference in the mean VAS scores was statistically significant at all measurement time-points after the procedure (*p* < 0.001). The mean VAS score in the first 24 h after treatment was 4.38 ± 1.00 in patients who underwent RFA with simultaneous cement injection. The differences between mean VAS scores obtained at the first, third, and sixth months after the procedure were statistically significant (*p* < 0.001). The VAS scores were 2.94 ± 1.04 and 2.44 ± 1.61 1 and 3 months after treatment, respectively, and 2.31 ± 1.42 6 months after treatment. An 82.4% reduction in pain was observed in all patients in the RFA + VP group and 52.7% of patients showed a significant improvement in the QoL. Moreover, the mean pre-treatment Oswestry Index (to assess the QoL) was 78.50% in the RFA + VP group, which improved to 14.2% after treatment (Table 2, Fig. 4).

The comparison of pre-treatment and 6-month post-treatment results of the Oswestry Disability Index revealed significant improvements in social life, walking ability, personal care, level of pain, and sleeping function in all patients. Tenoxicam and tramadol hydrochloride were discontinued 48 h after the procedure in patients who had required them before the procedure. The need for tenoxicam significantly decreased in both groups by the end of the first week.

Between-group comparisons revealed a significant decrease in pain in all patients from both groups after the first 3 months and a significant improvement in the QoL, with a lower degree of pain interfering with daily activities. There was no statistically significant difference. Between group comparisons, a significant difference was observed in VAS scores at the end of the 6th month. However, eight patients in group 1 required analgesics at the end of the third month.

Twelve patients died due to systemic problems during the follow-up (6 patients were group 1, 6 patients were group 2).

## Discussion

Metastatic lesions that spread to the spine can cause severe pain, spinal fractures, and neurological problems due to nerve root and spine compression by the tumour mass [7]. Several treatment alternatives for spinal metastases including radiotherapy, chemotherapy, isotopic therapy, bisphosphonate therapy, pharmacotherapy, RFA, and palliative surgery can be used [8]. The choice of treatment depends on the histopathology of the primary tumour, neurological function before treatment, number of involved vertebrae, vertebral level, site of the osteolytic lesions in the spinal body, degree of intraspinal diffusion, disease severity, and the patient’s general condition.

Narcotic analgesics are the first-line pharmacotherapy for pain control in patients with metastatic lesions; however, they can cause extreme drowsiness, constipation and nausea. Previous studies have reported that palliative radiotherapy is highly beneficial in alleviating metastasis-related pain [11, 12]. However, a 57% recurrence rate (of pain) was reported 15 weeks after the end of radiotherapy [9]. Approximately 40% of patients did not benefit from a second round of radiotherapy [13]. Reconstruction surgeries such as surgical decompression, pedicular screws, and corpectomy-cage placement can be performed in patients whose life expectancy exceeds 6 months. However, the complication rate is as high as 20–40%, and systemic complications such as surgical wound area infection, pneumonia, and urinary tract infections were observed in several patients [10].

Pain is the most common finding in patients with spinal metastasis, with a consequential reduction in mobility and deterioration in the QoL. Approximately 30–50% of patients with cancer experience pain, and excruciating pain disrupts the QoL in 75–90% of patients with advanced-stage cancer [9]. Bone metastasis-related pain is triggered by osseous destruction induced by osteoclasts, which are the principal bone resorption cells of the body. Biochemical factors and cytokines released from the periosteum and tumour cells also contribute to osseous destruction [14]. The pain caused by vertebral involvement is dull and stable and progressively increases, exacerbated by movement of the extremities [13, 15]. Radiation and/or chemotherapy, surgery, and use of opioids and other analgesics are common for pain control in patients with spinal metastasis. However, the QoL is extremely poor owing to intolerable pain in these patients. All patients in our study had a history of increasing analgesic use at least 2 months before the procedure.

While Goetz reported that analgesics use decreased significantly6 months after treatment in 41 of 43 patients treated with RFA [16]. Zhao determined a significant reduction in analgesics use 6 months after the implementation of RFA in 34 patients with metastases [17].

In our study, tramadol hydrochloride was discontinued 48 h after the procedure in groups 1 and 2, while the need for tenoxicam, another analgesic used at the end of the first week, significantly decreased. Group 1 patients had increased pain at the 3-month follow-up, and analgesic use increased before the procedure. This situation was attributed to the increase in the degree of collapse in the affected vertebra.

VP has also been reported to facilitate safe and rapid pain reduction in patients with cancer with spine involvement and increased patients’ ability to walk and perform daily activities [18]. The skeleton is stabilized with the application of VP after RFA, thus preventing periosteal deformation and pain [19]. RFA and PMM injection can be combined to reduce pain and improve the QoL. The advantage of performing RFA before PMM injection is increased control for PMM distribution, which can be useful in posteriorly located lesions [20]. Moreover, the spread and displacement of tumour cells are prevented by the ablation shell barrier, which is applied during RFA. RFA can also cause intravertebral venous plexus thrombosis, and subsequently reduce the risk of PMM leakage. Liu et al. reported that the combination of RFA, a minimally invasive intervention used for treating metastatic spine lesions, with percutaneous VP was particularly beneficial in reducing the incidence of fracture, risk of pain and surgery, and improving the QoL [7].

Lane reported a reduction in the pain scores in some patients treated with combined RFA and VP [20]. Gronemeyer reported a significant reduction in pain and disability in patients treated with RFA and VP [21]. In our study, PMM injection was applied to the vertebrae with metastatic lesions after successful RFA in 40 patients. In all of these patients, a significant decrease in pain scores and a significant increase in quality of life were found at the end of six-month controls. After the follow-up, no patient was found to have fracture development in the spine, and cord compression after tumor spread.

The reported rate of serious complications for percutaneous VP is low (< 10%); however, one study reported a PMM leakage rate of 81% visualised using CT [22]. Barragan-Campos reported 42 cases of PMM leakage cases from amongst 159 percutaneous VP procedures [23]; however, only 2 patients had serious complications. Furthermore, Nakatsuka reported that 4 patients developed hemiplegia and radiculopathy after RFA + VP, which was performed under CT fluoroscopy guidance [24]. In our study, PMM leakage occurred in 4 (25%) of 40 patients who underwent VP, but no serious complications were observed (Fig. 2).

All the aforementioned complications occur in cases where the tumour invades the vertebral cortex, and consequently, VP is contraindicated in these patients [25]. On the other hand, the combined procedure is safe in patients without posterior cortex and pedicular invasion. Shimony et al. [25] reported their successfully performing VP in patients with metastasis-related compression fractures but without posterior cortical deterioration, and successful pain control in 82% of patients, without any serious complications. Our study protocol achieved a successful pain control rate exceeding 80% 6 months postoperatively.

Goetz reported a reduction of at least 2 points in the pain scores in 95% of patients after RFA treatment [16]. The reduction in pain levels was the highest in the first week, with a rate of 41%, with significant reductions in the opioid requirement in the 8^th^and 12^th^weeks [16]. In our study, significant improvement was observed in patients with refractory pain caused by spinal metastasis. According to the VAS assessment, the mean decrease in pain was 3.3 points after 72 h, 5.3 points in the 1^st^week, 5.7 points in the 1^st^month, and 6.7 points in the 6^th^month.

## Conclusion

In many patients who develop spinal metastases, minimally invasive techniques should be preferred because of systemic problems and general condition deterioration. While pain control was successful in the first 3 months in both the RFA and RFA + VP groups, more successful results were obtained in the RFA + VP group after 3 months. RFA combined with VP is superior in treatment of spinal metastasis with respect to pain reduction, analgesic-consumption reduction, and tumour spread compared to use of RFA alone after 3 months. This procedure significantly reduced patients’ dependence on ‘drugs of last resort’ and wider tumour spread. None of the treated patients had a collapse in the treated vertebrae or tumor spread that continued during the 6-month follow-up period, and associated cord compression and neurological damage.

## Data Availability

The data can be accessed at any time from the archive records of Afyonkarahisar Health Sciences University Faculty of Medicine hospital. Due to the laws in our country, we cannot provide access to patient views. We can present all patient images as files. The datasets generated and/or analyzed during the current study are not publicly available due to patient rights laws, but are available from the corresponding author upon reasonable request.

## Abbreviations

RFA: Radiofrequency ablation
VP: Vertebroplasty
CT: Computed tomography
MR: Magnetic resonance
QoL: Quality of life
PMM: Polymethylmethacrylate
VAS: Visual Analogue Scale
